# Robot-Assisted Surgery in the Treatment of Gynecological Carcinoma and Malignancies: Introduction to the da Vinci Robotic Surgery System

**DOI:** 10.7759/cureus.43035

**Published:** 2023-08-06

**Authors:** Arnav Goel, Soumya Pamnani, Ashish Anjankar

**Affiliations:** 1 Medicine, Jawaharlal Nehru Medical College, Datta Meghe Institute of Higher Education & Research, Wardha, IND; 2 Biochemistry, Jawaharlal Nehru Medical College, Datta Meghe Institute of Higher Education & Research, Wardha, IND

**Keywords:** laparoscopy, conventional laparoscopy, disease-free survival, massive invasive suregery, food and drug administration, robot-assisted surgery

## Abstract

Robotic surgery is a surgical intervention that was developed from traditional manual surgeries because of the intrusive procedures it uses. It is now accomplished in hospitals worldwide, and comprehensive programs for the application of technology in the management of gynecological cancer are being developed. Robotic surgery should be straightforwardly compared with manual and traditional laparoscopy to see if the higher indirect costs are justified by some improvements in patient studies. This paper aims to evaluate the procedure of robotic surgery and its implementation in gynecological cancer to verify its safeness, practicability, and effectiveness. A higher chance of infections is usually in classical surgery, particularly in comparison to laparoscopic or robotic surgery. Surgical and hospital stay are much less with any of these new technologies than the aforementioned; however, the drawbacks are the scarcity of robot systems, their high price, and the realization that it is only appropriate in learning institutions with infrastructure and highly skilled surgeons. In conclusion, tissue engineering constitutes a significant discovery and approach for treating gynecological cancer with improved methods than some other types of traditional surgery, and it will likely become dominant technology shortly.

## Introduction and background

In this growing world, both population and technology are expanding rapidly. The use of radiotherapy, chemotherapy, or a combination of the two in the treatment of gynecologic cancer is an ever-evolving field [1]. Laparoscopic surgery has transformed the concept of manual surgery over the past three decades [1]. Since then, improved technology, cameras, and energy sources have enabled surgeons to undertake more intricate surgeries that were previously exclusively performed using laparotomies [2]. The early clinical success of robotic surgery in the diagnosis of symptoms or signs has urged more gynecologic doctors to investigate it. The focus is on the application of robotic surgery in the treatment of cervical, endometrial, and ovarian cancers in this article [3].

Robot-assisted surgery (RAS) is now becoming extremely prevalent in gynecological oncology. The specialist guides a robot that is appended to the patient via trocars comparable to those seen in laparoscopic surgery and robotic surgery. Gynecologic oncologists discovered early on that laparoscopic procedure was connected with fewer surgical comorbidities and a faster recovery time [4]. To address these concerns, minimally invasive surgery techniques, including endoscopic surgery, and, more recently, RAS have emerged. In the past few years, reports on the outcomes of RAS for uterine cancer and selected cases of ovarian cancer patients have become available [4]. Robotic surgery has worked in a number of women, including those who are morbidly obese, in poor health, or who have chronic conditions [5].

Elderly individuals have a higher rate of adverse prognostic variables, and age itself is a risk factor to consider when selecting therapy. In fact, more aggressive and advanced malignancies are frequently identified in this group of patients. In the majority of instances, surgery is the usual treatment [6]. The presence of multiple medical conditions, which increases the risk of surgical complications, is the most important factor in the management of elderly patients [7]. As a result, it is complicated to achieve the greatest oncological outcome possible through extensive surgery while also reducing pre, para, and postoperative problems and improving recovery durations [8].

## Review

Methodology

**Figure 1:**
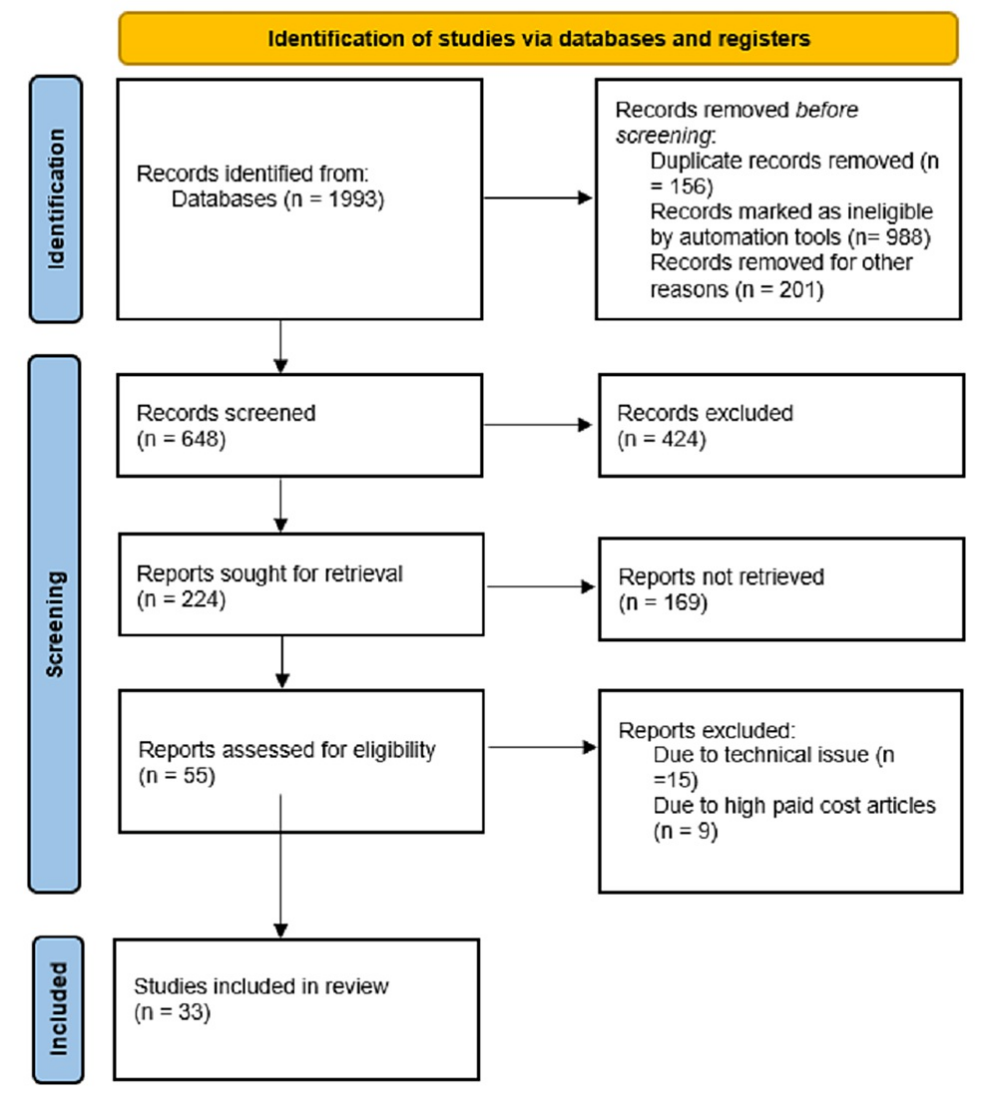
PRISMA flowchart for search PRISMA: Preferred Reporting Items for Systematic Reviews and Meta-Analyses.

We searched clinical trials and have taken statistical data from well-renowned and authentic journals and databases such as PubMed, Google Scholar, and Cochrane Library. Keywords such as "gynecological malignancy," "robotic surgery," "da Vinci System," and "uterine carcinoma" were searched in the medical database. We included only those articles that were in the English language. We excluded all articles that were in different languages and had old data and information. If multiple reports from the same study have been published, the most recent one was chosen for assessment. Only review articles were taken into account. The PRISMA (Preferred Reporting Items for Systematic Reviews and Meta-Analyses) flowchart is given below in Figure 1.

Reviews of the da Vinci System

The da Vinci Surgical System (Intuitive Surgical, Sunnyvale, CA) is a robot that is used in microsurgery. Many instruments are used with the da Vinci medical system to do robotic-assisted surgery [9]. People often make mistakes when they hear the word "robotic." Robots are not used to do surgery. With da Vinci, surgeons use the tools that they help guide with a computer for performing surgery [10]. The da Vinci System flexes and rotates the instruments while performing the procedure. The relatively small-handed instruments can move more easily than a human finger [11].

The da Vinci vision system also provides three-dimensional (3D) high-definition views of the surgical area that is highly magnified. Surgeons can operate through one or a few small incisions due to the instrument's size [12]. The da Vinci System includes a surgeon's gamepad in the very same space as the patient, and also a patient side bucket with three to four intriguing robotic arms controlled by the gamepad. The arms can be used as scalpels, scissors, bovies, or graspers, and they can hold objects [10]. Intuitive Surgical, the system's manufacturer, has been chastised for bypassing FDA approval through a process called "premarket confirmation.” In addition, the company is at risk of providing limited training and enabling healthcare professionals to cut down on the number of monitored actions required before such a doctor can use the scheme without guidance [13]. There are over 1,700 da Vinci Systems in hospitals around the world. The da Vinci procedure has been performed on more than 38.5 million patients worldwide. Procedures using the da Vinci robot are used in a variety of specialties, including cardiac, urologic, gynecologic, pediatric, and general surgery [14].

There are four types of da Vinci systems: (i) Si surgical system, (ii) X surgical system, (iii) Xi surgical system, and (iv) Sp surgical system [15].

Working

In the working of the modern method of conventional laparoscopy, the robotic arms have more mobility, allowing for easier suturing and complicated dissection with 3D vision [15,16]. Several recent research studies have found that robotic surgery is greater than traditional surgical procedure systems of internal bleeding, hospitalization, and complication rate, as well as, more importantly, disease-free survival [17]. However, there are still a surprisingly large number of possible trials to confirm the advantages and safety of gynecological RAS. Additionally, the cost-benefit comparison of RAS versus open surgery, particularly laparoscopy, in terms of postoperative outcome is still ambiguous [18]. By reviewing current published evidence on gynecological oncology, the purpose of this review study is to settle some of the existing disagreements [19]. The early clinical success of robotic surgery in the therapies of symptoms or signs has compelled more gynecologic oncologists to recognize it. We focus on how robotic surgery benefited gynecological cancer patients [20].

Advanced Elements of Robotic Surgery in the Treatment of Gynecologic Cancer

In significant ways, laparoscopic surgery differentiates from robotic surgical techniques [21]. A two-dimensional camera is used in traditional laparoscopy, and images are projected onto monitors near the surgeon in the operating room. A webcam and rigorous devices are positioned through epigastric port facilities and directly controlled by the surgeon at the surgical bedside, and surgery is conducted through five to 12-millimeter perforations [22]. As a result, the majority of patients who benefit from a minimally invasive procedure to treat gynecologic cancer is small. The gynecologic oncologist sits at a console apart from the trauma patient surgery, thanks to robotic platforms [13]. Gynecologic oncologists are interested in performing cancer surgeries on a minimally invasive robotic device even though the surgeon is no longer at the patient's bedside due to the 3D photonics, mastery offered by wrist-like equipment rotation, a slight decrease in surgeon hand tremor, and motion scaling [23]. A surgical advancement process called "minimally invasive surgery" has enabled minimizing the visibility of the infected area. The da Vinci Surgical System is a surgical robotics with a virtual 3D actuality visual acuity system that provides a greater profile in laparoscopic surgery where health professionals cannot physiologically see the affected area [24]. In 2000, the Food and Drug Administration approved the technology for clinical use [25].

Expenses of Robotic Surgery for Diagnostic and Therapeutic Practices

Automaton gynecological surgery is more expensive than traditional laparoscopic procedures. Economic data supporting or refuting emulation surgery are still missing from journals on the relative costs of oncologic robotic surgery [26]. When compared to Great Britain, Singapore, and other countries, the expense of robotic treatment in India is considerably lower. In India, the cost of a robotic surgery starts at INR 142,000. The estimated price of robotic surgery is INR 455,000 [27]. For full comment, the data are not relevant. Health and marketing researchers are investigating the financial implications of robotic surgery for diagnostic and therapeutic procedures. Implementing a robotics program for oncology services in post-secondary cancer centers may not allow for the possibility of starting with simple cases. Robot-assisted laparoscopy is costly, but in high-volume centers with high-volume surgeries, it can be cost-effective. When fully utilized and with price competition, surgery can be made affordable [10].

Discussion

We had the opportunity to analyze several gynecological treatments as well as the role of robotic-assisted laparoscopy in this study. The robot enables the surgeon to overcome some technological issues by minimizing vibrations and providing 3D vision and devices that can be precisely maneuvered in a full 360° [28]. Otherwise, the multiport robot approach yields excellent surgical results but leaves patients with many scars. Surgical scars should not be regarded as a "cosmetic concern," but rather a representation of the influence body image has on the patient, always reminding them of the malignancy [29]. Data from the articles that are chosen from PubMed also claim that the robotic surgical method can yield satisfactory treatment outcomes in patients with early-stage cervical cancer while having a low complication rate [27]. The use of the da Vinci robotic surgery instrumentation in gynecology has broadened the indications for minimally invasive surgery. The da Vinci Robot System features a 3D, elevated field of view, as well as a unique approximately 360° "wrist" range-of-motion flexibility, and close-up view of pelvic anatomy for precise surgical treatments [30].

**Figure 2:**
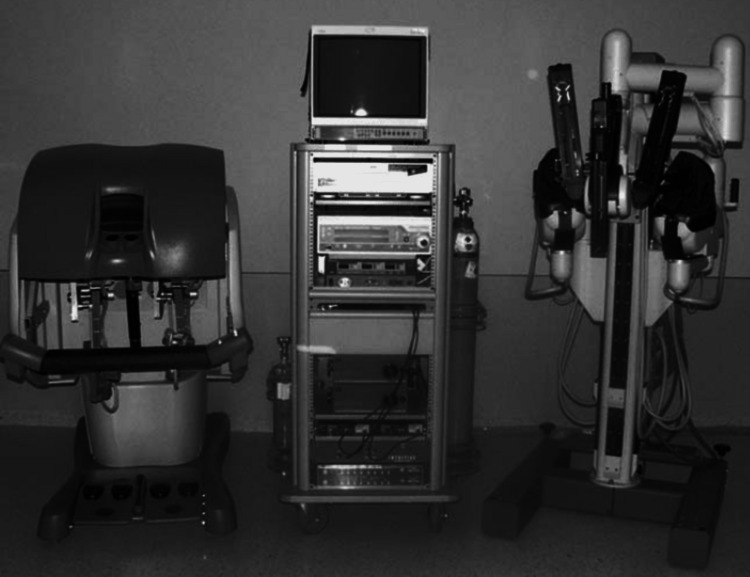
The da Vinci Surgical System Image under CC-BY license contributed by T. Schmid. Source: [32].

As a result, with training and surgeon expertise, robotic surgery appears to give an advantage in the removal of gynecological tumors. We propose more research into the cost and effect of RAS in gynecologic cancer [31]. Additional robotic surgery may enhance prognosis in advanced cervical cancer patients with recurrent or persistent illness after concomitant chemoradiotherapy. However, more prospective data are needed to properly describe the long-term survival of robotic surgery for cervical cancer [1]. Figure 2 shows the da Vinci Surgical System [32].

## Conclusions

Even though preliminary results have been offered over and over again after trying to suggest successful surgical techniques for women with gynecologic malignancies, people are still worried about how well surgery works. In research studies, RAS has led to better results, like a lower death rate. Robotic systems are used in almost every hospital to treat gynecologic cancers, but more study needs to be done on the technical aspects of surgical care. Additionally, cost comparisons between robotic surgery and conventional surgery are being made. The high costs and lack of adequate instruments could be a major barrier to robotic surgery's further expansion. But the effectiveness of the system is more than that of the manual surgery system. Exuberance and caution are appropriate when interpreting the robotic data due to its minute features of handling. At the time, the use of a robotic system in gynecological oncologic surgery provides significant benefits but it is not justified. The field of oncology is quickly accepting robotic-assisted surgery. Despite the lack of randomized controlled trials, the new technology appears to be useful and effective, with comparable oncological consequences in this population of patients. Many studies have examined the efficacy of the robotic system, thus many people are choosing robotics as their career option. Studies have also proven that operative time will decrease using the technology. The issue remains that the low number of techniques available limits access to this system but robotic surgery as a supplementary therapy for developed cancer patients with symptomatic disease after multiple simultaneous chemoradiotherapy may improve prognosis. So, the whole article depicts the power of the robotic system, and we the public evolving toward modernization should also accept modern technology.
